# Epstein-Barr virus and its association with disease - a review of relevance to general practice

**DOI:** 10.1186/s12875-019-0954-3

**Published:** 2019-05-14

**Authors:** Anders Fugl, Christen Lykkegaard Andersen

**Affiliations:** 1 The Copenhagen Primary Care Laboratory (CopLab) Database, Section of General Practice and The Research Unit for General Practice, Department of Public Health, University of Copenhagen, Copenhagen, Denmark; 2 Department of Hematology, Copenhagen University Hospital, Rigshospitalet, Copenhagen, Denmark

**Keywords:** Epstein-Barr virus. Infectious mononucleosis, Hepatitis, Splenic rupture, Airway compromise, Lymphoproliferative cancer, Multiple sclerosis, Rheumatoid arthritis, General practice, Family practice

## Abstract

**Background:**

General practitioners encounter the vast majority of patients with Epstein-Barr virus-related disease, i.e. infectious mononucleosis in children and adolescents. With the expanding knowledge regarding the multifaceted role of Epstein-Barr virus in both benign and malignant disease we chose to focus this review on Epstein-Barr virus-related conditions with relevance to the general practitioners. A PubMed and Google Scholar literature search was performed using PubMed’s MeSH terms of relevance to Epstein-Barr virus/infectious mononucleosis in regard to complications and associated conditions.

**Main text:**

In the present review, these included three early complications; hepatitis, splenic rupture and airway compromise, as well as possible late conditions; lymphoproliferative cancers, multiple sclerosis, rheumatoid arthritis, and chronic active Epstein-Barr virus infection. This review thus highlights recent advances in the understanding of Epstein-Barr virus pathogenesis, focusing on management, acute complications, referral indications and potentially associated conditions.

**Conclusions:**

Hepatitis is a common and self-limiting early complication to infectious mononucleosis and should be monitored with liver tests in more symptomatic cases. Splenic rupture is rare. Most cases are seen within 3 weeks after diagnosis of infectious mononucleosis and may occur spontaneously. There is no consensus on the safe return to physical activities, and ultrasonic assessment of spleen size may provide the best estimate of risk. Airway compromise due to tonsil enlargement is encountered in a minority of patients and should be treated with systemic corticosteroids during hospitalization. Association between lymphoproliferative cancers, especially Hodgkin lymphoma and Burkitt lymphoma, and infectious mononucleosis are well-established. Epstein-Barr virus infection/infectious mononucleosis as a risk factor for multiple sclerosis has been documented and may be linked to genetic susceptibility. Chronic active Epstein-Barr virus infection is rare. However, a general practitioner should be aware of this as a differential diagnosis in patients with persisting symptoms of infectious mononucleosis for more than 3 months.

## Background

Epstein-Barr virus (EBV) is a double-stranded DNA virus belonging to the Herpes family and the primary cause of infectious mononucleosis (IM), a common infection worldwide with a lifetime prevalence of 90% [1]. Symptoms of IM, so-called glandular fever usually manifest after an incubation period of four to seven weeks, and include fever, lymphadenopathy and pharyngitis. The vast majority of IM cases are self-limiting with an excellent prognosis, but in rare cases acute complications such as splenic rupture, hepatitis and severe tonsil enlargement with airway obstruction are encountered [2].

EBV has also for long been known to be an implicate in other conditions than IM. A simple literature search produces an extensive list of associations derived from case-reports through large epidemiologic studies such as lymphoproliferative disorders, head and neck cancer, breast cancer, systemic lupus erythematosus, vitamin D deficiency, chronic fatigue syndrome, thyroid disorders, rheumatoid arthritis (RA), multiple sclerosis (MS) as well as other autoimmune disorders. Concerning malignant disease alone, it has been estimated that EBV is associated with close to 200,000 malignancies worldwide each year, which has led to increasing interest in a vaccine against EBV infection [3, 4].

General practitioners (GPs) encounter the vast majority of patients with EBV-related disease, i.e. glandular fever in children and adolescents. They diagnose millions of cases yearly, give advice concerning management including restriction of physical activity and alcohol consumption and in some cases, refer patients to secondary care due to complications of the infection. Furthermore, GPs are prompted by their patients regarding risk of potentially associated conditions when infected with EBV. Therefore, GPs need to be able to recognize signs of IM, the possible differential diagnoses, evaluate the risks of complications and provide evidence-based recommendations for the individual patient.

With the expanding knowledge regarding the multifaceted role of EBV in both benign and malignant disease we chose to focus this review on EBV-related conditions of relevance to the GP. This review will discuss IM and three acute complications to IM; hepatitis, splenic rupture and airway compromise. Furthermore, we will address late associations to EBV/IM which the GP may be consulted about; lymphoproliferative cancers, MS, RA and chronic active Epstein-Barr virus infection (CAEBV).

## Material and methods

A PubMed and Google Scholar literature search was performed. We used PubMed’s MeSH terms IM, Epstein-Barr virus, human herpesvirus 4, HHV-4, mononucleosis, glandular fever, lymphoma, lymphoproliferative disorder/cancer, lymph node cancer, multiple sclerosis, MS, disseminated sclerosis, rheumatoid arthritis, RA, hepatitis, hepatic function, liver insufficiency, liver failure, respiratory insufficiency/failure/distress, hypoxia, airway compromise/obstruction and splenic rupture. We then screened abstracts to assess whether the search results were of relevance for the focus of this review. Systematic reviews, meta-analyses and Cochrane reviews were prioritized. Case-reports were included as citations for uncommon outcomes and outliers to the general consensus. Articles focusing on EBV-associated IM were given priority over other IM-causing pathogens. Same priority was given to articles focusing on immunocompetent individuals and young adults/adults over children. As the number of relevant articles increased so did the number of relevant references and keywords which refined our literature search on PubMed, and numerous relevant articles were presented through the ‘Similar articles’ tab on PubMed. This approach produced a pool of 96 relevant research articles including one umbrella review, six systematic reviews (including one Cochrane review), three meta-analyses, 36 reviews and 11 case reports, with publishing span from 1992 to 2019. Contradicting results and alternative perspectives on the subject were noted and presented.

## Main text

### Infectious mononucleosis

#### Pathogenesis

90% of IM cases are caused by EBV with the remaining 10 % caused by cytomegalovirus (CMV), human herpesvirus 6, herpes simplex virus type 1 and human immunodeficiency virus (HIV) [5]. EBV is found in saliva and is therefore transmitted through coughing, the sharing of food, kissing (hence the layman’s term ‘kissing decease’), etc. Low amounts of EBV may be detected in saliva throughout life in infected individuals. Peak levels are seen during the acute phase if the infection [6], however, a study has shown that patients may be highly contagious up to 180 days after the onset of symptoms and maybe even past that [7]. After successful transmission, EBV infects epithelial cells and resting B-cells in the oropharynx, starts replicating and thereby spreads throughout the body. This process represents the incubation period, which lasts about six (four to seven) weeks [8] and leads to an activation of cytotoxic T-lymphocytes (CTL) and natural killer cells. The activation of CTL in primary infection leads to cell-mediated immune response causing the clinical presentation of IM. However, when EBV infects B cells, the virus is able to restrict its expression of genes to nine proteins (out of approximately 100) thereby avoiding recognition of CTLs, and thus resulting in latent infection in the affected B-cells. Different patterns of these expressed so-called latency proteins have been described and seem to associate to different EBV-associated lymphoproliferative conditions such as Hodgkin Lymphoma (HL), Burkitt Lymphoma (BL) and post-transplant lymphoproliferative disorder (PTLD) depending on the expression pattern, please refer below. Reactivation of EBV may also occur in later life in the immunocompromised as seen after transplants, due to immunosuppressive medication, acquired immune deficiency syndrome (AIDS), etc. [9].

#### Clinical presentation

Whether an individual develops IM primarily depends on the timing of exposure to the virus. Young children rarely develop clinical signs of IM, whereas up to 70% [10] of adolescents and adults will present with the classical symptomatic triad of about two to four weeks of fever, pharyngitis and cervical lymphadenopathy with lymphocytosis [1]. The risk of severe symptoms is positively correlated to the age of the patient at time of primary infection [11]. This is of importance since debuting patient age has increased in the last past 15 years, thereby rendering more individuals at risk of severe IM [11]. White tonsillar exudates, sometimes even covering the tongue may be seen and distinguish IM from the more spotted coverings seen in bacterial tonsillitis [6]. Palatal petechiae have been reported in up to 50% of cases [6]. Hepatosplenomegaly and some degree of hepatitis are also common findings. In approximately 75% of cases there is a subclinical increase in alanine aminotransferase (ALAT) and about five to 10 % may present with jaundice due to diffuse damage to the liver parenchyma [8]. Splenomegaly may not be noticeable upon physical examination, but ultrasonic assessment of splenic size during acute IM has shown that all patients develop splenomegaly in varying degrees [12]. Patients with IM are therefore routinely advised to limit physical activity for at least 1 month after the onset of symptoms, however, there is yet to be developed a method of predicting the risk of splenic rupture [13]. Thus the “classic” patient with uncomplicated IM may be summarized with the following presentation; fever, pharyngitis, cervical lymphadenopathy, lymphocytosis, palatal petechiae and hepatosplenomegaly and with elevated ALAT.

#### Diagnosis

Though patients with IM often present with the aforementioned triad of symptoms, a GP should always consider the array of illnesses that can cause IM-like symptoms. A thorough history of the patient should elucidate recent travel- and sexual history, contact with animals, previous medical problems and family history of disease. An excellent review from 2007 concerning the diagnostic process for IM-like disease, has summarized conditions that may mimic IM, and that should be considered depending on the medical and personal history of the patient [5]. An algorithmic approach to the patient at this point is recommendable. Pregnancy, intravenous drug use or men having sex with men should prompt the GP to be even more alert. In pregnancy, serological testing for CMV and toxoplasmosis should be undertaken in addition to EBV. The latter two groups should be tested for HIV as well [6].

**Fig. 1:**
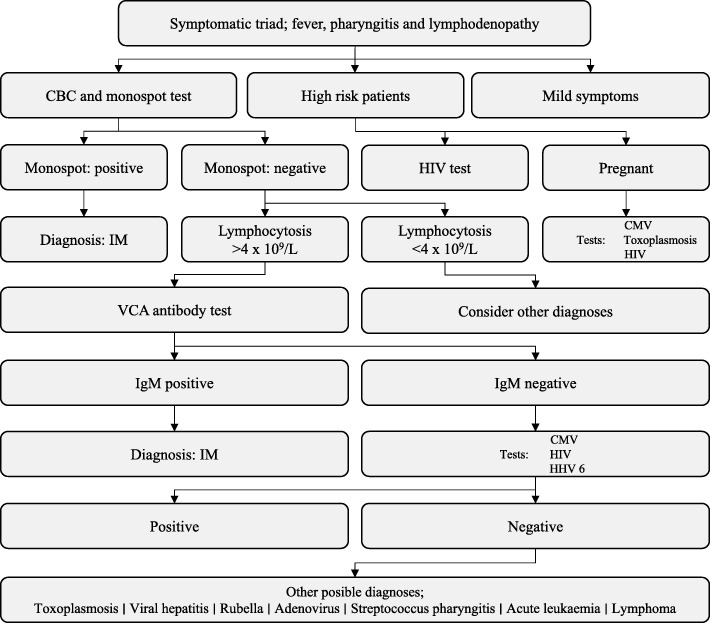
Algorithm for diagnosing IM. Adapted with permission from Lennon et al. [6]

If a patient does not classify as high risk, as defined above, the next step is blood testing (heterophile antibody test (Monospot test)) as well as a complete blood count (CBC). About 85% of patients with EBV-related IM, will have a positive heterophile antibody test, but the amount of heterophile antibodies may not be above the limit of detection within the first week of symptoms. This phenomenon can result in a false-negative rate of 25% in this period. However, a heterophile antibody test, with a sensitivity of 63–84% and a specificity of 84–100% [14], is still considered the best initial diagnostic test for EBV-related IM, due to its low cost and fast turn-around time. If a patient is truly heterophile antibody-negative, the GP should bear in mind that some 10 % of patients with IM are consistently heterophile-negative. In these cases, the EBV viral capsid antigen (VCA) IgG and IgM antibody test is of diagnostic value in the acute phase [5]. Additional testing for EBV nuclear antigen (EBNA) antibodies may help to distinguish between acute and past infection [15]. VCA and EBNA antibody tests offer higher sensitivity and specificity, but come at a higher price and take longer to analyze. In case of a heterophile antibody- and VCA-IgM-negative patient, a CBC may be used for further diagnostic workup. A diagnostic algorithm for use in primary care has been summarized in a recently published clinical review (Fig. 1) [6].

#### Treatment

There is currently no specific treatment for IM. Management focuses on symptomatic relief and patients are therefore treated with simple analgesics, antipyretics, hydration and rest. Of interest, a systematic review from 2002 advises against prolonged rest due to possible deconditioning. Deconditioning may cause prolonging of symptoms including fatigue and increases recovery time and patients should be allowed out of bed as soon as they feel fit enough [16]. There has been controversy surrounding a possible link between acute viral infections, such as IM, and chronic fatigue syndrome (CFS), but at present, it is unlikely that an acute EBV infection leading to IM can cause CFS by itself [6]. Antivirals for IM has been proposed for some time. However, there has not been any firm evidence to support this notion. A Cochrane review from 2016 concluded that the effectiveness of antiviral agents (acyclovir, valomaciclovir and valacyclovir) in acute IM is uncertain and that the quality of the evidence was low [17]. Thus, at the moment treatment should focus on supportive care which in severe cases may require hospitalization for intravenous fluids due to dehydration or monitoring for impeding respiratory failure.

### Early complications to infectious mononucleosis

#### Hepatitis

As mentioned, parenchymal injury of the liver tissue is common with about 75% of patients exhibiting a two to three fold increase in ALAT during the acute phase of infection [8] with levels returning to normal after about three weeks [18]. Cholestatic liver disease and chronic hepatitis due to EBV are rare complications, but have been described in the literature [19–21]. The hepatic changes are therefore usually transient and self-limiting, but cases of liver failure with fatal outcomes - even in immunocompetent patients - have been reported [22, 23] which is why it is advisable to order liver tests upon diagnosis and during monitoring of patients with more severe IM.

#### Splenic rupture

Splenic rupture, as a result of splenomegaly and altered splenic architecture [24], is probably the most feared and acute complication to IM. It is, however, a very rare complication with an incidence varying from 0.1–0.2% [25] to 0.5% [26], and with most cases occurring within the first 3 weeks after diagnosis. This is supported by a study of the ultrasonic assessment of splenic size during IM in athletes, which showed a peak increase 12.3 days after onset of symptoms. The study also demonstrated a predictable rate of regression of spleen size of about 1 % per day after the peak size [12]. The risk of splenic rupture is anecdotally highest in patients who are involved in contact sports like martial arts, football, hockey, etc., and activities with high intraabdominal pressure like weightlifting. Athletes are withheld from practice and competition during this phase of the infection, which may have implications for their academic- and athletic career, especially in countries like the US where athletic scholarships can determine whether a student goes to college. Much literature has therefore focused on the safe return to sports. There seems to be a current consensus that return to non-contact activities can commence 3 weeks after symptomatic onset [25]. On the other hand, recommendations regarding the return to contact sports vary, ranging from three to 5 weeks to 6 months [27]. Multiple attempts have been made to make clinical guidelines, but none have been accepted as general practice. One of the more sophisticated attempts was made by McKeag et al who focused on time from onset of symptoms and criteria such as fever, lymphadenopathy, bilirubin- and liver enzyme levels and absence of splenomegaly verified radiographically [27]. Still, several cases of spontaneous splenic rupture have been reported [28] and splenic rupture should therefore be suspected in all patients presenting with acute abdominal- or chest pain and with confirmed- or suspected IM.

#### Airway compromise

Cervical lymphadenopathy is a hallmark in IM and involves the nasopharyngeal and palatal tonsils. Swelling has been estimated to cause airway compromise in 1–3.5% of IM cases [2] – and more common among younger children [29]. Symptoms include stridor, cyanosis and tachypnea and should lead to immediate admittance and treatment. Systemic corticosteroids are used in patients with risk of airway compromise and show significant results within 12–36 h if treated aggressively. Early treatment with systemic corticosteroids may therefore limit the need for surgical intervention in severe cases [29]. Tracheotomy has only been used in extremely severe (and exceptionally described) cases, where treatment with systemic corticosteroids have failed [29]. Selected use of acute tonsillectomy in patients who have not responded satisfactorily to systemic corticosteroids has been proposed by some groups [30, 31], but has been a topic of debate, due to the conflicting data on the risk of bleeding. A study from 2005 reported that 27 of 205 (13%) patients experienced post-operative bleeding, with a total of 36 cases with a bleeding episode (18%). These results were not supported by those advocating for selected use of acute tonsillectomy [30, 31] and it seems that more research on this topic is warranted. Of importance, treatment with systemic corticosteroids is only warranted in an emergency setting, in the case of acute complications such as impending airway compromise. Systemic corticosteroids should not be instituted for symptomatic relief as it does not affect the incidence of complications nor rates of admission or length of hospitalization [32].

### Late complications to EBV/IM

#### Lymphoproliferative cancers

Lymphoproliferative cancers are the most well-established late-onset complication to IM and have been investigated in several different cohorts since the 1970s. A Scandinavian study from 2003 found an increased risk of EBV-positive HL in young adults, with an odds ratio (OR) of 2.7 (95% confidence interval (CI): 1.2 to 6.0), and a median incubation period of 4.1 years with a peak risk after 2.1 years after primary infection [3]. A British study from 2009 found similar results in two different cohorts. Both showed elevated risk of HL with rate ratios of 3.2 (95% CI: 1.2–7.0) and 6.0 (95% CI: 2.4–12.5), respectively [33]. An Italian multicenter case-control study from 2000 also observed an increased risk, with an age-adjusted OR of 4.4 (95% CI: 1.1–16.6). This study also demonstrated an increased risk of non-Hodgkin lymphoma (NHL), with an age adjusted OR of 4.0 (95% CI: 1.4–11.8) [34]; results that could not be confirmed by the Scandinavian study, however. Overall, published data seem to agree on a strong correlation between IM and HL where results concerning NHL in general are more conflicting. On the other hand, for certain NHL subtypes a strong correlation seems to exist. The carcinogenic properties of EBV was first hypothesized in 1964, when EBV was observed in cultured tumor cells from patients with BL in tropical Africa. This highly aggressive type of B-cell lymphoma has since been strongly correlated to EBV. There are three subtypes of BL; endemic, sporadic and immunodeficiency-associated, where the endemic subtype has shown the strongest correlation with EBV [35]. EBV status does not affect the treatment regime however, which consists of high-intensity chemotherapy and anti-CD20 monoclonal antibody therapy in fit patients and results in remission in > 85% of cases [36]. EBV has also been associated with diffuse large B-cell lymphoma (DLBCL) in 10 % of cases among immunocompetent patients. EBV-positive DLBCL seems to affect primarily elderly patients and is now termed EBV(+) DLBCL, not otherwise specified (NOS). This condition has shown to impair prognosis as these patients tend to respond poorly to conventional therapy [36].

As previously discussed, IM may lead to airway compromise as the mentioned lymphoproliferative cancers may themselves cause airway obstruction. However, this is not within the scope of this review nor relevant to a GP.

#### Multiple sclerosis

There is a substantial controversy concerning the causative role of EBV in the development of MS. It seems clear, however, that there is an association. A meta-analysis from 2006 showed that the risk of MS in EBV-negative individuals was close to zero whereas the incidence rate rose in EBV-positive individuals without clinical IM. Furthermore, the risk of MS was 2.3 times higher in individuals with a late EBV infection and history of IM than in EBV-positive individuals without a history of IM. The study also found that the incidence rate peaked around the age of 25–30 and declined to almost zero around 60 years of age [37]. A large umbrella review published in 2015, studying 44 different proposed risk factors for MS found similar results, with strong epidemiological evidence for IM as a risk factor for MS [38]. A study of the correlation of IM and initial symptoms of MS from 2017 supports a causative relationship between IM and MS and state that the rate of which MS develops could depend on genetically susceptibility to EBV infection as well as time of infection, with postpubescent infection being critical for the initiation and rapid development of MS [39]. Despite the published data, it is a challenge to prove underlying mechanisms. Humanized mouse models have been studied, but graft-versus-host reactions have been major confounders in these studies [40]. A study from 2013 on this controversy summarized the key issues in the debate: There is currently no consensus as to whether patients with MS has EBV-infected B-cells in the CNS [41]. This is further complicated by the “Hit-and-Run” hypothesis, which describes how EBV is able to ‘escape’ EBV-induced malignant cells after infection with no trace of virus itself [42]. However, it is not clear if this hypothesis only applies to malignant cells, or if EBV has a general ability to make a clean ‘escape’ from all types of infected cells including B-cells, which serve as essential implicants in MS [43]. If there indeed exits a causal link between EBV and MS, this would support that vaccination against EBV could have the potential to eliminate MS as one study suggests or, that insuring early life exposure to EBV could decrease the risk of developing MS, as individuals with a history of symptomatic IM exhibit a 2.3 times higher risk than EBV-positive individuals without a history of IM as mentioned above [37]. There is yet to be marketed such a vaccine, although a phase 2 placebo-controlled randomized clinical trial reported a reduced incidence of IM by 78%, however, the vaccine did not protect against EBV infection [44].

#### Rheumatoid arthritis

A causal link between RA and IM has been proposed alongside several other autoimmune diseases. However, a large systematic review and meta-analysis from 2015 found no significant association. The authors concluded, however, that several of the included studies had limitations and that more data on the subject was needed [45]. At present, no data demonstrate a clear association between EBV and RA, but it is worth acknowledging the increasing amount of literature suggesting diverse roles of EBV in autoimmune disease.

#### Chronic active Epstein-Barr virus infection

CAEBV is rare and mostly limited to Japan and East Asia, but has gained international attention due to an increase in cases worldwide. Although rare, a GP should be aware of this as a differential diagnosis in patients with IM-symptoms persisting for more than 3 months, after exclusion of IM, autoimmune disease and immunodeficiency disorders (congenital or acquired) [46]. Further diagnostic work should be performed by a specialist in haematology.

## Conclusions

The expanding roles of EBV in both benign and malignant disease bear witness of a viral species with diverse effects on the human body. GPs encounter the vast majority of patients exhibiting symptoms indicative of the most common EBV infection, IM. GPs of today therefore need to be aware of not only the classical early complications to IM, but also late complications and potential associated conditions as patients are entitled to evidence-based risk estimates of such outcomes.

Upon initial evaluation of a patient with symptoms suggestive of IM an algorithmic approach is recommended to ensure correct diagnosis and proper risk stratification. Treatment should focus on symptomatic relief and rest; however, prolonged bed-rest is discouraged as deconditioning may prolong symptoms and recovery time. Hospitalization can be necessary in severe cases of dehydration and impeding respiratory failure. Hepatitis is a common and self-limiting early complication to IM, but liver tests should be monitored in more symptomatic cases as fatal events have been reported. Also, splenomegaly may be noticed upon physical examination and consequently increases the risk of splenic rupture. Splenic rupture is rare, however, and most cases are seen within 3 weeks after diagnosis of IM and may occur spontaneously. Currently, there is no consensus on the safe return to physical activities, and ultrasonic assessment of spleen size may provide the best estimate of risk. Airway compromise due to tonsil enlargement is encountered in a minority of patients (1–3.5%) and is more common among younger children. Such patients should be admitted to pediatric wards, where early intervention with systemic corticosteroids limits the need for surgical intervention and thereby the risk of post-operative bleeding, infection and prolonged hospitalization.

The level of evidence for the late complications varies, but the association between lymphoproliferative cancers, especially HL and BL, and IM are well-established. Epstein-Barr virus infection/IM as a risk factor for MS has been documented and may be linked to genetic susceptibility, however the pathological mechanisms are yet to be described. Regarding RA and EBV, there is no significant association. CAEBV is rare, but a GP should be aware of this as a differential diagnosis in patients with persisting IM-symptoms for more than 3 months.

Due to the high prevalence of EBV-infected individuals throughout the world, and the known and proposed late complications to IM, preventive measures should be prioritized, ideally through vaccination. Current vaccination studies have shown success as far as preventing IM in 78% of cases, but does not prevent the infection with EBV. Future research will hopefully shed light on costs, benefits, side-effects and target-groups as well as determining whether the prevention of EBV-infection in general - or the prevention of IM in particular - is the primary goal. Furthermore, future research should focus on substantiating the known- and proposed associations and causations in order to contribute to the understanding of the pathogenic mechanisms of EBV.

## Abbreviations

AIDS: Acquired Immune Deficiency Syndrome
ALAT: Alanin Aminotransferase
BL: Burkitt Lymphoma
CAEBV: Chronic Active Epstein-Barr Virus
CBC: Complete Blood Count
CFS: Chronic Fatigue Syndrome
CI: Confidence Interval
CMV: Cytomegalovirus
CNS: Central Nervous System
CTL: Cytotoxic T-cell
DLBCL: Diffuse Large B-Cell Lymphoma
EBNA: Epstein-Barr Virus Nuclear Antigen
EBV: Epstein-Barr Virus
GP: General Practitioner
HIV: Human Immunodeficiency Virus
HL: Hodgkin Lymphoma
IgG: Immunoglobulin G
IgM: Immunoglobulin M
IM: Infectious Mononucleosis
MS: Multiple Sclerosis
NHL: Non-Hodgkin Lymphoma
NOS: Not Otherwise Specified
OR: Odds Ratio
PTLD: Post-Transplant Lymphoproliferative Disorder
RA: Rheumatoid Arthritis
VCA: Viral Capsid Antigen
